# Molecular Pathways in Pulmonary Arterial Hypertension

**DOI:** 10.3390/ijms231710001

**Published:** 2022-09-02

**Authors:** Aangi J. Shah, Mounica Vorla, Dinesh K. Kalra

**Affiliations:** 1 Department of Internal Medicine, School of Medicine, University of Louisville, Louisville, KY 40202, USA; 2 Division of Cardiology, School of Medicine, University of Louisville, Louisville, KY 40202, USA

**Keywords:** pulmonary arterial hypertension, molecular pathogenesis, classification, pathobiology

## Abstract

Pulmonary arterial hypertension is a multifactorial, chronic disease process that leads to pulmonary arterial endothelial dysfunction and smooth muscular hypertrophy, resulting in impaired pliability and hemodynamics of the pulmonary vascular system, and consequent right ventricular dysfunction. Existing treatments target limited pathways with only modest improvement in disease morbidity, and little or no improvement in mortality. Ongoing research has focused on the molecular basis of pulmonary arterial hypertension and is going to be important in the discovery of new treatments and genetic pathways involved. This review focuses on the molecular pathogenesis of pulmonary arterial hypertension.

## 1. Introduction

Pulmonary hypertension (PH) is a chronic condition characterized by a progressive pulmonary arterial pan-vasculopathy with consequent elevation in pulmonary arterial pressure and downstream hemodynamic and mechanical abnormalities in the pulmonary vessels and right ventricle. The first World Symposium on Pulmonary Hypertension (WSPH) in 1973 arbitrarily defined PH as a resting mean pulmonary arterial pressure (mPAP) ≥ 25 mm Hg, as measured by right heart catheterization [1]. However, in a 2016 retrospective review of 21,727 veterans undergoing right heart catheterization, Maron and colleagues noted higher clinical risk and mortality hazard emerging at mPAP of approximately 19 mm Hg [2]. Subsequently in 2018, Kovacs et al. studied 1187 heathy individuals and noted that supine mPAP at rest was 14.0 ± 3.3 mm Hg, with an upper normal limit, i.e., two standard deviations above the mean, of 20 mm Hg [3]. Additional studies on patients with systemic sclerosis also noted an increased risk of disease progression and elevated mortality in patients with borderline mPAP, defined as 21–24 mm Hg [4,5]. In the most recent Proceedings of the 6th World Symposium on Pulmonary Hypertension, the hemodynamic threshold for defining pulmonary hypertension was lowered to a mPAP of >20 mm Hg [6], with further expansion of the definition of PH to include pre- and post-capillary PH.

## 2. Epidemiology

The global incidence of PH as around 1% and as may be as high as 20–50% in elderly subjects, with 80% of the burden described in the developing countries harboring high rates of pulmonary infections [7]. Many registries, dating back to the 1980s, have detailed the incidence, prevalence, natural history, and prognosis of pulmonary arterial hypertension (PAH) [8]. Data from eleven major registries show that the incidence of PAH is 2.0–7.6 cases per one million adults per year and prevalence is 10.6–26 per million adults [8] (Table 1). However, with the widespread use of Doppler echocardiography permitting early diagnosis and advent of targeted therapies for PAH, the epidemiology of PAH has changed in the modern era. Notably, the average age of PAH diagnosis cited in the earliest NIH registry in the 1980s was 36 years. Newer registries show a shift in demographics such that more older patients are getting affected (≥45 years). Notwithstanding this observation, mortality statistics have trended positively over the last two decades. Median survival has risen from 2.8 years in 1980 to 6 years in the contemporary datasets [8,9]. Registries have also demonstrated a female predominance (4:1 in REVEAL registry, 2:1 in the UK, Ireland, and French Registries), but survival is poorer in affected males [10].

While the most common subgroup of PAH is idiopathic PAH (iPAH), accounting for 50–60% cases of PAH in USA and Europe, PAH secondary to connective tissue disorders is a close second [7,9]. Patients with systemic sclerosis without interstitial lung disease have a 12% prevalence of PAH, with survival deteriorating once interstitial lung disease develops [11,12]. Some of the infectious etiologies responsible for PAH are HIV, schistosomiasis, and the novel coronavirus 2019 infection (COVID-19). Despite the introduction of highly active anti-retroviral therapies, the burden of PAH in HIV positive patients is as high as 0.5% worldwide. HIV as the etiology of PAH is highest in sub-Saharan Africa, with prevalence exceeding 10% in certain regions [7]. In these regions, HIV related PAH is seen in 0.5 per 1000 individuals, which is much higher than the worldwide prevalence of PAH [13]. *Schistosoma species* infection, endemic to sub-Saharan Africa and Brazil has also been implicated in development of PAH via translocation of worm eggs from the portal circulation to the pulmonary vasculature with ensuing granulomatous changes, culminating in a immune-mediated vasculopathy and fibrosis that are responsible for PAH [14]. Prevalence of PAH secondary to schistosomiasis is over 50% of the local population in the endemic regions, with an even higher risk in patients with concomitant hepatic or splenic disease [7,11]. Reports of COVID-19 infection leading to pulmonary vasculopathy, described by post-mortem histological evaluations revealed thickened pulmonary walls, and suggested a vascular injury phenotype [15].

PH secondary to chronic thromboembolic pulmonary embolism (CTEPH) is categorized as group 4 PH. The 6th WSPH defined CTEPH as mPAP ≥ 20 mm Hg and pulmonary capillary wedge pressure ≤15 mm Hg diagnosed by right heart catheterization in the setting of flow limiting pulmonary artery thrombi/emboli [1]. Studies have reported an average annual incidence of CTEPH as 4%, higher than prior estimates of 0.1–0.5% [16,17], but this still might be an underrepresentation given that most patients are not evaluated for PH following an episode of pulmonary embolism and a majority of patients with CTEPH do not recall prior venous thromboembolism events [18]. The FOllow-up after aCUte pulmonary emboliSm (FOCUS) study was a multicenter observational cohort study which followed 1017 adult patients with confirmed acute symptomatic pulmonary embolism for a period of 2 years. The estimated 2-year cumulative incidence of pulmonary hypertension in this study was 2.3% (1.2–4.4%) [19]. CTEPH has been reported to have equal incidence in both males and females, unlike iPAH which is more common in females [18]. The burden of CTEPH is higher with increasing age, with the greatest prevalence noted in the sixth decade of life, as would be expected with increasing incidence of venous thrombosis and embolism with aging [18].

**Table 1:** Pulmonary arterial hypertension epidemiology registries (Adapted with permission from McGoon et al., 2013 [8]. Copyright 2013 American College of Cardiology Foundation, Elsevier Inc.) PH—Pulmonary hypertension; CHD—Congenital heart disease; CTD—Connective tissue disease; CTEPH—Chronic thromboembolic pulmonary hypertension; HPAH—Heritable pulmonary arterial hypertension; IPAH—Idiopathic pulmonary arterial hypertension; MAI—Million adult inhabitants; MI—Million inhabitants; NA—Not available; NIH—National Institutes of Health; PAH—Pulmonary arterial hypertension; PHC—Pulmonary hypertension connection; SMR—Scottish morbidity record; Ent—Entire study population; Inc—incident or newly diagnosed patients; Pre—Prevalent or previously diagnosed patients; CTEPH—chronic thromboembolic pulmonary hypertension; PTE—Pulmonary thromboembolism; PE—Pulmonary embolism. * Survival calculation only from IPAH and CTD-PAH patients.

Registry/Study	Study Cohort	Study Design and Time	No. of Patients	Incidence/Prevalence of PAH	Survival %			
					1 Year	2 Years	3 Years	5 Years
U.S. NIH	IPAH	Prospective, 1981–1985	187	NA	NA	NA	NA	NA
U.S. PHC	Group 1 PH, age > 18 years	Retrospective, 1982–2004; prospective, 2004–2006	578	NA	84	NA	67	58
Scottish-SMR	Group 1 PH (IPAH, CHD-PAH, CTD-PAH), age 16–65 years	Retrospective, 1986–2001	374	PAH, 7.6/26 cases/MAI; IPAH, 2.6/9 cases/MAI	NA	NA	NA	NA
French	Group 1 PH, age > 18 years	Prospective, 2002–2003	674	PAH, 2.4/15 cases/MAI; IPAH, 1.0/5.9 cases/MAI	Ent 87 Pre 88 Inc 88	Ent 76 Pre 79 Inc 65	Ent 67 Pre 71 Inc 51	NA
Chinese	IPAH and HPAH	Prospective, 1999–2004	72	NA	NA	NA	NA	NA
U.S. REVEAL	Group 1 PH	Prospective, 2006–2009	3515 (age > 3 months)	PAH, 2.0/10.6 cases/MAI; IPAH, 0.9 cases/MAI	85	NA	68	57
Spanish	Group 1 PH and CTEPH, age > 14 years	Retrospective, 1998–2006; prospective, 2007–2008	PAH, 866; CTEPH, 162	PAH, 3.2/16 cases/MAI; IPAH, 1.2/4.6 cases/MAI	NA	NA	NA	NA
UK	IPAH, HPAH, anorexigen-associated PAH	Prospective, 2001–2009	482	1.1/6.6 cases/MI	79 *	68 *	57 *	NA
New Chinese Registry	Group 1 PH, age > 18 years	Prospective, 2008–2011	956	NA	NA	NA	NA	NA
Mayo	Group 1 PH	Prospective, 1995–2004	484	NA	81	NA	61	48
Compera	IPAH, age > 18 years	Prospective, 2007–2011	587	NA	NA	NA	NA	NA
Gall et al. [20]	CTEPH epidemiological databases	Retrospective literature review, up to June 2014	NA	USA & Europe: 3–5 cases/100,000 Japan: 1.9/10,000	NA	NA	NA	NA
Pepke-Zaba et al. [16]	CTEPH	Prospective, 2007–2009	679	History of PE: 74.8% History of thrombophilic conditions: 31.9% History of splenectomy: 3.4%	NA	NA	NA	NA
Korkmaz et al. [21]	PTE	Retrospective January 2006–October 2008; Prospective November 2008–November 2009	325	Incidence after first episode of PE: 4.6% Incidence after 12 months following PTE attack: 80%	NA	NA	NA	NA
FOCUS study [19]	Acute symptomatic PE (adult)	Prospective, 2014–2018	1017	2-year cumulative incidence of 2.3% (1.2–4.4%)	NA	NA	NA	NA
Otero et al. [22]	Acute symptomatic PE	Prospective, 2003–2004	744	Incidence of mPAP ≥ 50 mm Hg was 8.3% at 36 months	NA	NA	NA	NA
Poli et al. [23]	Acute symptomatic PE	Prospective, 2010	287	Incidence after first episode of PE: 0.4%	NA	NA	NA	NA
Pengo et al. [24]	Acute PE	Prospective, 2004	223	3.8%	NA	NA	NA	NA
Miniati et al. [25]	Suspected acute PE	Prospective, 2006	834; 320 patients with confirmed PE	1.3% in group with confirmed PE	NA	NA	NA	NA
Becattini et al. [26]	First acute PE	Prospective, 2006 (3-year median follow up)	259	1%	NA	NA	NA	NA
Dentali et al. [27]	First acute PE	Prospective, 2009	91	8.8%	NA	NA	NA	NA
Klok et al. [28]	Acute PE	Prospective, 2001–2007	866	Incidence for all-cause PE: 0.57% Incidence for unprovoked PE: 1.5%	NA	NA	NA	NA

## 3. Classification of Pulmonary Hypertension

The earliest classification of pulmonary hypertension was presented by Dr. Paul Wood in 1950s, based on his own clinical observations and noteworthy works by Castleman and Bland, and Edwards [11]. This classic work has often been lauded as ‘unsurpassed’ and was remarkable for the time it was published, sans the current-day technology. Wood classified pulmonary hypertension on the premise of physiology as five distinct types: passive, hyperkinetic, obliterative/obstructive, vasoconstrictive, and polygenic or mixed [29].

### 3.1. Hemodynamic Classification of Pulmonary Hypertension

The newer epidemiological databases encompassing worldwide populations have robustly supported earlier studies which advocated for a cutoff of mPAP ≥ 19 mm Hg as being elevated, because this has important prognostic implications [3]. This led to the 6th WSPH to lower mPAP threshold to >20 mm Hg, offsetting the earlier concerns of overdiagnosis and overtreatment of PH. The chances of over-diagnosing are effectively diminished with accurate diagnosis established by right heart catheterization [30]. Hemodynamic classification delineates PH into pre-capillary, isolated post-capillary, and mixed pre- and post-capillary PH, by defining a threshold value for pulmonary vascular resistance (PVR) and pulmonary capillary wedge pressure (PCWP), with the latter being indicative of the left ventricular end-diastolic pressure (LVEDP) in the absence of mitral valvular abnormality or any mechanical obstruction between the LV and pulmonary capillary network.

Pre-capillary PH is defined as mPAP > 20 mm Hg, PVR ≥ 3 Woods Units (1 WU = 80 mm Hg/L/minute) and PCWP ≤ 15 mm Hg. This designation distinguishes PH resulting from disorders in the pulmonary arterial bed. Clinical-hemodynamic combinations possible for pre-capillary PH are clinical groups 1, 3, 4, and 5, as described below. Post-capillary PH is defined as mPAP > 20 mm Hg, PVR < 3 WU and PCWP > 15 mm Hg. This is seen in groups 2 and 5 PH (group 2 is due to left heart disease) [1]. Mixed pre- and post-capillary PH is mPAP > 20 mm Hg, PVR ≥ 3 WU and PCWP > 15 mm Hg and carries a worse prognosis [31]. The major causes of mixed pre- and post-capillary PH are again, LV dysfunction, valvular heart diseases, heart failure with preserved ejection fraction—however, the precapillary component develops after a longstanding period of postcapillary pulmonary venous hypertension due to superimposed adverse remodeling in the pulmonary arterioles. The latter does not occur in isolated pure group 2 PH.

### 3.2. Clinical Classification of PH

The 6th WSPH classification was published in 2018 and divided pulmonary hypertension into 5 clinical groups with several sub-groups. The purpose of developing a clinical classification was to divide PH based on underlying hemodynamic characteristics, pathophysiology, clinical manifestations, and overall therapeutic management, recognizing however that some hemodynamic-clinical-prognostic correlations may not always be correlated based on such a schema.

The World Health Organization (WHO) has also published a classification detailing the functional tolerance in PH, as in Table 2.

## 4. Pathophysiology of Pulmonary Arterial Hypertension

As opposed to the systemic circulation, the pulmonary vascular anatomy is arranged in parallel circuits facilitating a low pressure, low resistance circuit with consequently, higher blood flow. A distinctive challenge for the pulmonary circulation is to transform high-pressure pulsatile right ventricular output into smooth low-pressure flow into the capillaries [33]. The sequential branching of pulmonary vessels also allows for maximizing the gas-exchange capacity at the alveolar-capillary interface.

The pulmonary arteries are, like the systemic circulation, divided into elastic, transitional and muscular arteries, based on the muscularity in arterial media and the presence of elastic lamina in the vessel wall. The basic structure of pulmonary arteries and arterioles consists of an outermost layer of adventitia, that is generally abutting the airway and alveolar surface on opposite ends, arterial media consisting of varying composition of elastic lamina and smooth muscle cells and innermost layer of non-fenestrated endothelial cells lining the vessel lumen [34]. Fibroblasts, in addition to some other interstitial cells, present in the arterial media are implicated in the fibrogenesis that underlies the pathologic vascular remodeling in PH. Given the inflammation in the disease process, pulmonary artery endothelium acquires an increasingly aberrant adhesive capacity with a dysregulated proliferative and anti-apoptotic growth constitution giving rise of in situ thrombosis [1].

Extension of Ohm’s law to fluid dynamics generates the hemodynamic relationship between flow, pressure and resistance as, the change in pressure (∆P) equals the product of resistance (here, pulmonary vascular resistance or PVR) and flow (here, mean pulmonary blood flow or Q), i.e., ∆P = PVR × Q. PVR is measured in Woods units which is mm Hg/L/min, named after Dr. Paul Wood. For clinical calculation, PVR is measured as the difference of mPAP and left atrial pressure. Pulmonary capillary wedge pressure (PCWP) is generally designated as a surrogate measure of left atrial pressure, given it is frequently unfeasible in clinical settings to measure the latter.

### Pathologic Remodeling in Pulmonary Vessels

**Figure 1:**
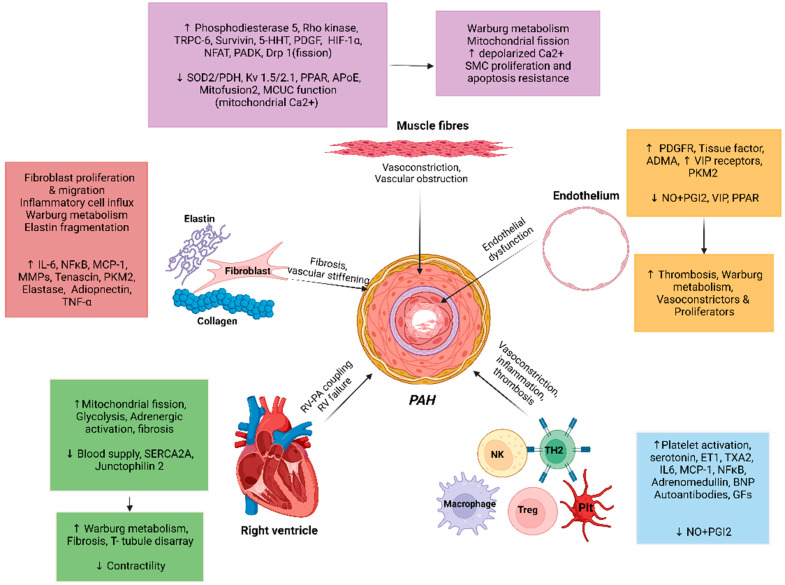
Pathogenesis of pulmonary artery hypertension. 5-HHT: 5 Hydroxy tryptamine; Apo E: Apolipoprotein E; ADMA: Asymmetric dimethylarginine; BMPR2: Bone morphogenetic protein receptor 2; BNP: Brain natriuretic peptide; Ca^2+^: Calcium; DNAMT: DNA methyltransferase; Drp-1: Dynamin related protein 1; ET1: Endothelin; HDAC: Histone deacetylases; HIF: Hypoxia inducible factor; IL: Interleukin; MCP-1: Monocyte chemoattractant protein-1; MCUC: Mitochondrial calcium uniporter complex; miRNA: Micro RNA; MMP: Matrix metalloproteinase; NFAT: Nuclear factor of activated T cells; NF-kB: Nuclear factor kappa light chain enhancer of activated B cells; NK: Natural killer cells; NO: Nitric oxide; PDGFR: Platelet derived growth factor receptor; PDGR: Platelet derived growth factor; PDH: Pyruvate dehydrogenase; PDK: Pyruvate dehydrogenase kinase; PGI2: Prostacyclin; PKM2: Pyruvate kinase M2; PPAR: Peroxisome proliferator activated receptor; SERCA: Sarco-endoplasmic reticulum Ca^2+^ ATPase; SERT: Serotonin transporter; SMC: Smooth muscle cell; SNP: Single nucleotide polymorphism; SOD: Superoxide dismutase; Th2: T helper cells; TNF: Tumor necrosis factor; TRPC: Transient receptor potential cation channel; T-reg: Regulatory T cells; TxA2: Thromboxane A2; VIP: Vasoactive intestinal peptide, ↑:Increased expression; ↓: Decreased expression.

**Figure 2:**
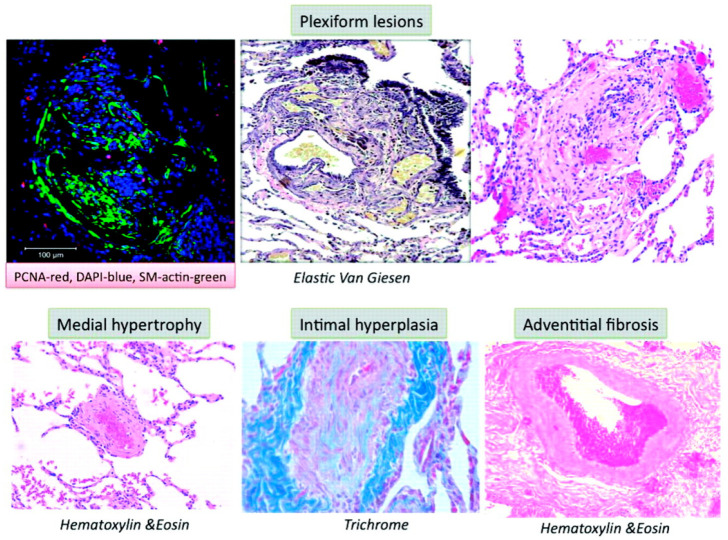
Pulmonary artery histology in pulmonary arterial hypertension (Reproduced with permission from Archer et al., 2010 [38]. Copyright 2010, Wolters Kluwer Health).

Multiple molecular mechanisms in concert with chronic environmental, metabolic, genetic, and hypoxic insults in PAH result in increased oxidant stress, inflammation, and dysregulated metabolism that culminate into pathologic remodeling of pulmonary vessels, primarily affecting the medium- and small-sized pulmonary arterioles. While the inceptive instigating injury may vary, all pathologic pathways eventually conclude in vasoconstriction, dysregulated smooth muscle, and endothelial cell proliferation, fibrogenesis and micro-thrombosis, with an ensuing rise in PVR (Figure 1) [35]. The pathologic changes seen in PAH are microvascular thrombosis, muscularization of distal pulmonary arterioles, and classic histologic finding of plexiform-heterogenous remodeling [11,36,37] (Figure 2) [38]. However, the pathophysiologic relevance of the latter is yet undefined [39]. The increased PVR and volume- and pressure-overload on right ventricle (RV) induces compensatory hypertrophy. The consequent elevation in RV end-diastolic volume and progressive tricuspid regurgitation sets forth a vicious cycle of pathologic RV adaptation. It has been noted that RV is a thin-walled and otherwise compact structure in comparison to LV and rather poorly suited for hypertrophy [40]. With increasing PVR, RV initially compensates by increasing its contractility, by up to 4–5-fold. However, with further PH progression, RV hypertrophy fails to compensate, and it is instead RV dilation that is attempted as a measure to ensure continued RV-Pulmonary artery (PA) coupling. This is associated with reduced stroke volume, and results in compensatory tachycardia to maintain the cardiac output [41]. These changes conclude with eventual RV-PA uncoupling and clinical manifestations of RV failure.

## 5. Molecular Pathways in Pulmonary Arterial Hypertension

While insight into some molecular pathways in PAH such as endothelin-1 dependent, prostacyclin-mediated, role of vascular calcium channels and nitric oxide driven pathways, have aided in development of newer targeted therapies for PAH with improved quality of life, the overall mortality in PAH has not significantly changed, with only modest improvement in functional and hemodynamic parameters. While all the aforementioned treatment avenues target to counteract vasoconstriction, it has been reported that less than ten percent of patients with PAH present a dominant vasoconstrictive pathophenotype [9].

In 2001, Newman et al. described the presence of a thymine-to-guanine transversion on the BMPR2 gene in 18 PAH families and presented a potential single gene insult as the basis for this disease [42]. However, it has been reported that less than thirty percent of the patients with PAH have single gene causative variants, thus allowing for numerous post-transcriptional mechanisms, with superimposed environmental insults to be involved in the overall presentation of PAH [43]. Better insights into the molecular pathogenesis of PAH are necessary for provide comprehensive understanding of the disease process and ascertain newer therapeutic targets for management.

### 5.1. Role of Nitric Oxide

**Figure 3:**
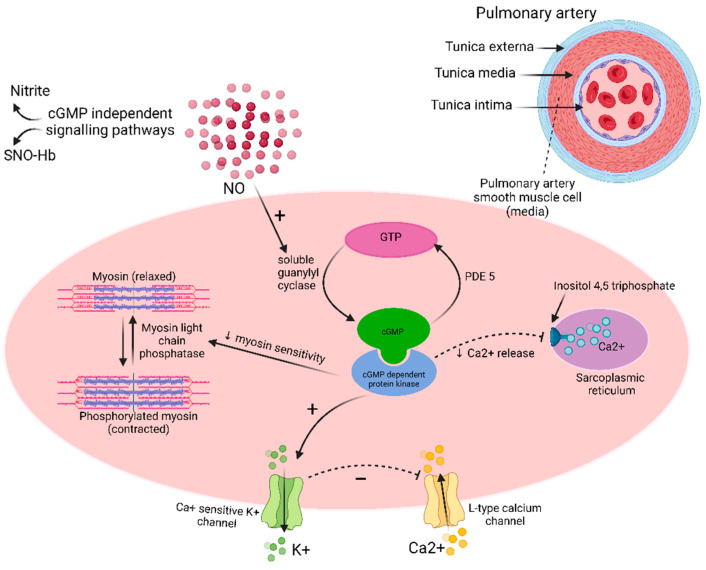
Role of nitric oxide in pulmonary arterial hypertension. SNO-Hb: S-nitroso-hemoglobin; NO: Nitric oxide; PDE: Phosphodiesterase; cGMP: Cyclic guanosine monophosphate; GTP: Guanosine triphosphate. “+” = stimulates, “−” = inhibits.

Nitric oxide (NO) is a signaling hormone synthesized in the endothelium by oxidation of L-arginine to L-citrulline to NO, in the presence of oxygen (O_2_), tetrahydrobiopterin (BH_4_), reduced nicotinamide adenine dinucleotide phosphate (NADPH) and NO Synthase (NOS) enzyme [44]. NO radical (NO˙) has local vasodilatory effects on the vascular smooth muscle cells via soluble guanylate cyclase (sGC) pathway and increased production of cyclic guanosine monophosphate (cGMP) (Figure 3). NO˙ activates sGC and catalyzes conversion of guanosine-5′-triphosphate (GTP) into cGMP. cGMP in turn activates cGMP-dependent protein kinase that activates calcium sensitive potassium channels and inhibits sarcoplasmic reticulum mediated calcium release. The overall decrease in the intracellular calcium inhibits myosin phosphorylation and the consequent vasoconstriction. cGMP regulates phosphodiesterases (PDE) as well [45]. NO also effects vasodilation independent of cGMP via S-nitrosylation or via oxidation to form nitrite to cause post-translational modification of proteins [46,47]. Other roles played by NO in vascular homeostasis involve regulation of vascular smooth muscle cell growth via inhibiting apoptosis, inhibition of platelet thrombosis [35], inhibition of vascular smooth cell proliferation, and inhibition of collagen production [47].

In PAH, decreased bioavailability of NO is one of the major pathogenetic mechanisms. Studies have shown decreased bronchoalveolar lavage levels of NO biochemical products, decreased exhaled levels of NO, and reduced whole body NO production in patients with PAH [45,47,48]. Experimental models have also reported that a defect in BH_4_ synthesis can result in pulmonary hypertension due to a decrease in NOS activity and subsequent deficiency of NO [49].

Data is conflicting regarding the variation in levels of expression of endothelial NOS (eNOS) in PAH. Earlier reports mentioned decreased eNOS expression in PAH [50], which is in contradistinction to later reports stating increased eNOS immunostaining in PH [51] and interestingly, augmented expression in the pulmonary artery plexiform lesions in PAH [52]. Some data state that dysfunctional eNOS [35] is the culprit, while others theorize that it is the reduced eNOS activity rather than total eNOS expression that is responsible for PAH [47].

NO has also been implicated in PAH pathogenesis in patients with iPAH or hereditary PAH due to BMPR2 (Bone morphogenic protein receptor) variants. Gangopahyay et al. demonstrated that in patients with BMPR2 mutations, BMP2 and 4 failed to stimulate eNOS phosphorylation with consequent low eNOS activity. Failure of eNOS mediated protein kinase activation led to the endothelial dysfunction and in the PAH pathophenotype [53].

### 5.2. Role of Prostacyclin

Prostanoids are compounds derived from plasma membrane lipids, which when acted upon by phospholipase A_2_ are converted into arachidonic acid or 5, 8, 11, 14-eicosatetraenoic acid. Arachidonic acid is further metabolized into paracrine hormones prostaglandins (PG) and thromboxane (TXA_2_), and leukotrienes (LT) via action of the enzymes cyclooxygenases (COX) and 5-lipoxygenases (5-LO), respectively. COX enzymes have two isoforms, COX-1 that is constitutively expressed in diverse cell and tissue types, including vascular endothelium. COX-2 is an inducible isoform present in vascular endothelium and expressed during inflammation. Arachidonic acid then, by the action of COX, is converted into TXA_2_ and prostacyclin (PGI_2_).

TXA_2_ is a potent vasoconstrictor and platelet aggregator, while PGI_2_ is a vasodilator and inhibits platelet aggregation [54]. It has been hypothesized that a skewed TXA_2_/PGI_2_ balance, with decreased PGI2 and exaggeration of TXA_2_ drives the vasoconstrictive endophenotype observed in PAH. Supporting observational data reports decreased PGI_2_ synthase mRNA in pulmonary capillaries and arterioles of PAH patients, increased TXA_2_ levels and sensitivity in animal models of hypoxia induced PAH and reduced urinary excretion of PG metabolites with increased urinary levels of TXA_2_ metabolites in PAH patients compared to healthy subjects [55,56,57]. Vascular shear stress, inflammation, hypoxia and TGF-β receptor genetic variants all play roles in inciting COX-2 activation [37].

The role of synthetic Prostanoids is well established in the treatment of PAH (intravenous epoprostenol and treprostinil, subcutaneous treprostinil, inhalational iloprost and treprostinil, and oral treprostinil and beraprost). Selexipag has also been shown to be beneficial in the treatment of PAH. Sitbon and colleagues conducted a phase 3 randomized double-blind, placebo-controlled trial with 1156 patients with PAH and randomized them to receive selexipag 1600 µg twice daily or placebo. The study reported lower risk of death from any cause or complications related to PAH in the selexipag group [58].

### 5.3. Role of Endothelin-1

Endothelin-1 (ET-1) is a vasoconstrictor molecule and a smooth muscle mitogen produced by the endothelium. ET-1 regulates its action by binding to ET_A_ and ET_B_ receptors. Activation of ET_A_ receptors by ET-1 activates both phospholipase C-β and increase inositol triphosphate, thus causing downstream increase in intracellular calcium which leads to vasoconstriction and a potent mitogenic response. In addition, ET-1 may also play a part in fibrogenesis via interaction with matrix metalloproteinases [59], smooth muscle hypertrophy, cellular proliferation, leukocytic activation and pronunciation of vascular adhesive phenotype. Endothelin is not stored, but rather synthesized in the endothelium and its gene transcription is regulated by the genes c-fos and c-jun, acute inflammatory agents, and nuclear factor -1. Micro-environmental factors altering ET-1 production include hypoxia, mechanical shear stress on vessel walls, oxidized LDL cholesterol, estrogen levels, inflammatory cytokines, and adhesion molecules [60]. Endothelin receptor antagonists (bosentan, ambrisentan and macitentan) are established therapy for PAH [61].

### 5.4. Role of Serotonin

5-hydroxytryptamin (5-HT or serotonin) is a neurotransmitter synthesized predominantly in the enterochromaffin neuroendocrine cells in the small intestine, colon, and appendix, but also in the endothelial cells of pulmonary artery. It has been reported that the enzyme tryptophan hydroxylase, present in the pulmonary endothelial cells and the first step in the serotonin synthesis pathway, is upregulated in PAH [11]. Serotonin binds to and activates G-protein coupled receptors on pulmonary artery smooth muscle cells, leading to decrease in adenylate cyclase and cAMP, culminating in vasoconstriction [62]. Via paracrine signaling, serotonin through its action on vascular smooth muscle and endothelial cells induces proliferation, mitogenesis, fibrogenesis and results in vasoconstrictive endophenotype reported in PAH. It is also through this mechanism that serotonin participates in the vascular remodeling observed in PAH [59]. Serotonin mediates the action through increased expression of serotonin transporter (SERT), which on binding with 5-HT activates downstream signal transduction via cellular kinases leading to transcription of pro-proliferative genes [63].

Augmented endothelial 5HT production has been noted in iPAH patients [64]. Similarly, elevated plasma serotonin levels with reduced platelet levels have been reported in patients with iPAH [65]. The existing literature is still controversial regarding the role of SERT gene mutations in the pathogenesis, but data supporting this has cited gain of function polymorphisms in SERT (SERT promotor L/S gene polymorphisms) in some patients—however, this was not observed uniformly in other patients with PAH [66,67]. Mice models have suggested aggravated PAH with increased SERT expression either globally or locally in pulmonary artery smooth muscle cells [68,69]. Additionally, they have also reported tempering of PAH endophenotypes with deletion of the SERT gene [70].

Studies have also shown increased incidence of PAH in patients taking dexfenfluramine (also known as anorexigenic PAH). Dexfenfluramine is an appetite suppressant that increases serotonin release from platelets and inhibits serotonin reuptake, causing elevated serotonin levels [71]. However, the causative role of serotonin plasma levels in PAH pathogenesis is debatable, as selective serotonin reuptake inhibitors (SSRIs) which also raise plasma serotonin levels have not been associated with PAH [72].

Serotonin can also act via other pathways such as the angiopoetin-1/Tie2 signaling pathway or by inhibition of BMP2 signaling via SMAD proteins [62]. Zhang et al. showed the role of microRNAs which by regulating serotonin production via SERT mediate the vascular proliferative endophenotype. Patients with PAH were noted to have lower levels of miR-361-3p and higher levels of pulmonary vascular resistance and mPAP. Serotonin treatment reduced miR-361-3p expression in human pulmonary artery smooth muscle cells while the over-expression of miR-361-3p was associated with suppressed cell proliferation, G1 cell cycle phase arrest and augmented apoptosis. SERT is a target for miR-361-3p action, as upregulation of SERT attenuated the effects of decreased miR-361-3p on serotonin induced pulmonary artery smooth muscle cell proliferation. The authors thus concluded that miR-361-3p may be a potential target for PAH therapies [73].

### 5.5. Role of Endothelial Dysfunction

The endothelium is a semi-permeable barrier lining the capillaries and arterioles, and it plays a critical role in pulmonary vascular homeostasis. A healthy pulmonary endothelial barrier is responsible for the modulation of growth, vascular tone, response to injury, differentiation and the overall flux of blood and cells. Endothelial dysfunction has a crucial pathogenetic role in PAH.

An imbalance between vasoconstriction and vasodilation is observed in PAH. Irrespective of the inciting factor, vasoconstriction, coupled with vascular fibrosis and endothelial cell proliferation lead to pathogenic remodeling that is characteristic of PH. Plexiform arterial lesions are a result of inflammation, fibrosis, and vascular remodeling, and may add to vascular wall thickening discerned in PAH. The instigating vascular injury has been noted to upregulate production of vasoconstrictive mediators such as endothelin-1 (ET-1), thromboxane A_2_ (TXA_2_), serotonin, and angiotensin-II, while downregulating the production of vasodilators, namely NO, adrenomedullin and prostacyclin (PGI_2_) [65]. Endothelial injury in PAH can be caused by many factors, hypoxia, environmental toxins, exaggeration of signaling molecules (VEGF, FGF, tyrosine kinases), inflammatory mediators (interleukins, cytokines, chemokines), and pathological shear stress on the vessels because of noncompliant hemodynamics [59]. However, the exact chronology of these changes is unclear, meaning that the endothelial dysfunctional phenotypes are seen to occur in concurrence to PAH changes as well preceding and succeeding them [36].

Vascular endothelial dysfunction also promotes a thrombogenic state, with patients with long-standing disease having a higher burden of micro-thrombosis. While there have been multiple reports of coagulopathies in patients with PAH (protein C and S deficiencies, von Willebrand factor dysfunction), the accurate pathologic role is unclear as most coagulation factors are acute phase reactants and emerging theories are increasingly emphasizing the role of inflammation in PAH pathogenesis [74]. Anticoagulation therapies in PAH have been controversial with some survival benefit with warfarin observed for patients with iPAH [35,74].

Endothelial cell proliferation is also observed in PAH pathogenic remodeling. Peroxisome proliferator-activated receptor-Υ (PPAR-Υ) expression has been observed to be reduced in patients with severe PAH while normal levels seen in patients with otherwise normal lungs but also in those with chronic obstructive pulmonary disease (COPD). In vitro studies have also shown decreased PPAR-Υ in the setting of fluid shear stress. The said evidence has led researchers to conclude that decreased PPAR-Υ expression in PH is a result of vascular shear stress [75]. Endothelial cell homoeostasis, regulation of cell cycle genes, ubiquitin-mediated DNA repair pathways, attenuation of VEGF-mediated endothelial dysfunction are all affected due to suppression of PPAR-Υ in PAH [36]. Studies in a hypoxia rat model noted reversal of severe PAH and vascular remodeling and alleviation of right ventricular failure with oral treatment with PPAR-Υ agonist pioglitazone [76]. Authors concluded that PPAR-Υ activation can regulate epigenetic and transcriptional changes responsible for pathogenesis of PAH.

“Master switch” theories postulate a single molecule as the principal factor responsible for a given endophenotype. Transforming growth factor-β (TGF-β) has been implicated to be the key molecule driving collagen deposition and subsequent fibrosis across different cell- and tissue-types [77]. TGF-β receptor-II activated by via canonical SMAD2/3 (TGF-β activin nodal branch) is the primary mechanism described to regulate gene transcription coding for collagen deposition. Extensive cross-interaction between TGF-β activin nodal branch and bone-morphogenic protein (BMP)-growth differentiation factor (GDF) branch in concert with multiple genetic and environmental factors is responsible for regulation of cell-differentiation and proliferation [78]. Over 300 mutations in BMP receptor 2 (BMPR2) gene have been observed in patients with PAH [79]. The BMPR2 genetic variant (T345G variant) has been identified in 70% of families with PAH and in around 10% of cases with sporadic iPAH [11]. BMPR2 is a type II receptor of TGF-β superfamily, and in recent studies, mutations in other members of TGF-β have also been identified [78].

However, master switch theories have been called ‘insufficient’ to comprehensively described the pathogenic fibrosis as they overlook the bio-functionality [77]. Network analysis studies by Samokhin and colleagues recognized neural precursor cell expressed developmentally down-regulated (NEDD9) as a critical pro-fibrotic mediator in PAH. Researchers collected various genes responsible for pathogenic and adaptive fibrosis, excluding genes associated with lung fibrosis, along with aldosterone regulated genes in the human vascular cells. The resulting genes are mapped on a consolidated human protein-to-protein interactome. Analyzing the resulting interactome using betweenness centrality (BC), authors recognized malignancy protein NEDD9 as an important aldosterone-mediated node responsible for pathogenic vascular fibrosis. Elevated levels of aldosterone have been recognized to induce oxidative stress in PAH. Aldosterone induced oxidant stress in pulmonary artery endothelial cells results in oxidation of Cys [18] in small mothers against decapentaplegic 3 (SMAD3), leading to impaired SMAD3-NEDD9 interaction and consequently impaired NEDD9 degradation. Thus increased NEDD9-Nk2 homeobox 5 (NKX2-5) and NKX2-5-COL3A1 complexes upregulate collagen III transcription, which in turn culminate in increasing cell stiffness in PAECs [77,80]. Identification of NEDD9 mediated cellular stiffness and fibrosis is an important TGF-β independent mechanism given that in the authors noted no changes in the signaling pathway with TGF-β ligand trap [77].

Endothelial to mesenchymal transition is a process by which endothelial cells phenotypically transform to acquire properties of mesenchymal cells. These endothelial cells have been observed to be responsible for accumulation of α smooth muscle cells expressing actin in pulmonary vascular lesions. The cellular plasticity is induced by TGF-β superfamily signaling via both canonical (SMAD2/3) and non-canonical pathways (ERK1/2 and p38 MAPK) which is a result of increased exposure to inflammatory mediators in PAH [81]. BMPR2 signaling alterations have also been reported to be responsible for endothelial to mesenchymal transition seen in PAH [82,83,84].

### 5.6. Role of Calcium and Potassium ion Channels

Calcium (Ca) plays a role in vasoconstriction and smooth muscle cell proliferation. Elevated cytosolic calcium level in pulmonary artery smooth muscle cells leads to activation of store operated calcium channels, resulting in further increase in calcium levels. The increased intracellular calcium levels also inhibit potassium channels, specifically voltage gated potassium channels Kv1.5 [9]. These potassium channels are also inhibited by hypoxia [59]. Downregulation of Kv.1.5, causes membrane depolarization, further calcium influx via voltage gated Ca channels (Ca_L_) and subsequent vasoconstriction via Ca-Calmodulin and myosin light chain kinase [59]. Such vasoconstriction is seen in the setting of hypoxia (hypoxic vasoconstriction) but also due to defects in the function or expression of Kv.1.5 channels [35]. In iPAH, transient receptor potential (TRP) channels 3 and 6 are found to be upregulated and are suspected to play a role in PAH pathogenesis through aberrant Ca signaling [59]. Calcium driven activation of nuclear factor of activated T-cells (NFAT), which is sensitive to Ca^+2^/Calcineurin, promotes an apoptosis resistant, cancer like endophenotype, by increasing expression of anti-apoptotic bcl-2 [85].

In addition, impaired mitochondrial calcium uptake via mitochondrial calcium uniporter (MCU) also plays a part in increased intracellular calcium and its associated downstream events as mentioned above. PAH endophenotype is observed in normal pulmonary artery smooth muscle cells with inhibition of MCU. Notably, micro-RNAs, especially miR-138 and miR-25, have been theorized to be responsible for MCU downregulation as well as its transcriptional regulator. Researchers have hypothesized miR-MCU complex as a potential therapeutic target for alleviation of PAH [86].

Impaired calcium sensitization mediated by rho-kinase is also a possible pathogenetic mechanism resulting in impaired calcium homeostasis and resulting hypoxic vasoconstriction seen in PAH. Recent studies have reported hyperactive RhoA/ROK (*Ras* homologue gene family, member A/Rho kinase) pathway in PAH [87,88]. There is emerging evidence of the role of rho kinase in PAH pathogenesis through promoting smooth muscle cell proliferation, inflammation, interaction with serotonin resulting in vasoconstrictive endophenotype, BMPR2 mediating signaling and promoting remodeling through its interactions with NFAT. Thus, rho kinase inhibitors too, are a potential therapeutic target in treatment of PAH [89].

### 5.7. Role of Mitochondrial Metabolic Dysfunction

Otto Warburg described the ‘Warburg effect’ in early 1920s as the metabolic shift to aerobic glycolysis seen in cancer cells. This has also been observed in PAH. Initially described only in pulmonary artery smooth muscle cells, Warburg effect has now also been observed in pulmonary endothelial cells and right ventricular myocytes in the setting of ischemia [9]. This metabolic shift from coupled oxidative glucose metabolism to uncoupled aerobic glycolysis occurs in abnormal cells resulting in suppression of mitochondrial respiration [80]. This is a consequence of suppression of oxidative glucose metabolism due to phosphorylation and subsequent inhibition of pyruvate dehydrogenase (PDH) by pyruvate dehydrogenase kinase (PDK), latter being upregulated in PAH [9]. Additionally, aerobic cytosolic glycolysis is also promoted in PAH via upregulation of glucose transporters on the plasma membrane and increased expression of a variant pyruvate kinase which is the concluding enzyme in the cytosolic glycolytic pathway. A normoxic upregulation of hypoxia inducing factor (HIF)-1 is also an associated factor in upregulating aerobic cytosolic glycolysis [81].

This departure from normal anerobic glycolysis, provides the abnormal cells with energy to thrive and fosters a proliferative, anti-apoptotic endophenotype. Zhang et al. demonstrated an increase in pyruvate kinase muscle isoforms 2/1 (PKM) ratio in PAH, which in turn promoted the metabolic dysfunction in the cells. They also reported reversal of glycolytic phenotype with normalization of PKM2/1 ratio using miR124 overexpression [90]. Dichloroacetate (DCA), has been identified as a potential therapeutic option in reversal of this metabolic phenotype. DCA inhibits PDK and is and has been used to treat congenital lactic acidosis in children [91]. DCA is a hypothesized treatment option for PAH and has been shown to reverse PAH in monocrotaline animal models [92].

Mitochondrial calcium uniporter (MCU) complex as mentioned above is downregulated in PAH. While mitochondrial PDH inhibition increases cytosolic Ca^2+^ levels, it lowers mitochondrial Ca^2+^ levels. The consequent low calcium state induces mitochondrial membrane hyperpolarization, which in concert with other factors such as elevated mitogen expression in PAH and reduced PPAR-Υ co-factor 1-α, induces a mitochondrial fusion/fission imbalance [9,93]. Dynamin related protein (Drp)-1 is the mitochondria fission mediator while mitofusion-2 is the fusion mediator. Pronounced drp-1 with negative regulation of mitofusion-2 results in mitochondrial fission out of sync with the cell cycle, witnessed in PAH as increased percentage of fragmented mitochondria in cytosol of pulmonary artery smooth muscle cells. Thus, drp-1 has also been implied as a potential therapeutic target to treat PAH [94]. miR-138 and miR-25 are proposed therapeutic targets to maintain the MCU level to promote reversal the metabolic dysfunction [86].

### 5.8. Role of Sex Hormones

Bone morphogenic protein receptor type II (BMPR2) mutations are the most identified genetic mutations in heritable PAH. Reduced BMPR2 expression has been noted to be estrogen dependent [67], with higher penetrance noted in females (40%) as compared to males (14%) [95]. This is also associated with pronounced proliferation seen in human pulmonary artery smooth muscle cells via phosphokinase signaling pathways [95]. Estrogen has been noted to reduce BMPR2 expression via its action on ΕR (estrogen receptor)-α [96]. Downstream metabolites such as 16α-hydroxyestrone have been implied in pulmonary smooth muscle cell hypertrophy and subsequent vascular remodeling in experimental models [97]. Estrogen action on ER-β receptors has been observed to protect against PAH phenotype [95].

Estrogen receptor inhibition via anastrozole has showed attenuation of PAH severity and improving BMPR2 signaling in female animal models but not in males [98]. In a small double blind randomized placebo controlled clinical trial, 18 patients with PAH were randomized to receive either anastrozole or placebo in a 2:1 ratio. The study showed improved 6 min walk distance in anastrozole group (+26 m) as compared to the placebo group (−12 m) [99].

Increased prevalence of metabolic syndrome is seen in females with iPAH, and studies have hypothesized that insulin resistance insulin resistance may be a risk factor for PAH, especially in females [100].

### 5.9. Role of Renin-Angiotensin-Aldosterone System

The role of angiotensin the pathogenesis of PAH has been supported in many preclinical and basic research studies. A hypoxic hypobaric rat model-based study showed a 50% increase in angiotensin converting enzyme (ACE) activity in remodeled pulmonary vessels and an associated increased ACE mRNA expression. ACE, principally located in the pulmonary endothelial cells, is responsible for conversion of angiotensin I to angiotensin II. Angiotensin II is the active hormone in the RAAS system, manifesting a vasoconstrictive phenotype via its action on AT1 receptors, while stimulation of AT2 receptors results in vasodilation [101]. Further on, ACE2 enzyme, is responsible for metabolism of angiotensin II to angiotensin-(1-7), which also brings about vasodilation via its action on G protein coupled receptors. Studies have shown a AT1 upregulation and associated vasoconstriction noticed in pulmonary artery smooth muscle cells. They also report a downregulation of AT2 and angiotensin-(1-7) [102]. Increase in ACE activity has been reported to occur not just in the pulmonary vascular anatomy. A 3.4-fold increase in ACE activity has been reported in the right ventricular myocardium in the setting of a fortnight of induced hypoxia [103].

The role of ACE inhibitors and angiotensin receptor blocker (ARBs) has been studied in the PAH. Captopril and losartan have been reported to reduce the mPAP and reverse RV myocardial hypertrophy in PAH animal models [104]. On similar lines, losartan has shown improvement in RV remodeling and fibrosis with subsequent improvement in RV hemodynamics and reduction in RV afterload [105].

Endothelin-1 when increased in PAH, is a major secretagogue for aldosterone [102]. Hypoxia rat models have demonstrated a 442% increase in plasma aldosterone levels and 183% increase in lung tissue aldosterone. This was seen in association with elevation of endothelin levels in these models. Aldosterone mediates endothelial cell dysfunction by interrupting endothelin (ET) B-receptor mediated activation of eNOS and consequently causing reduction in NO production. Aldosterone also augments oxidative stress in these settings. Spironolactone, a mineralocorticoid receptor antagonist, was reported to decrease the oxidative burden instigated by hyperaldosteronism and reinstate ET-B receptor functioning. In addition, Maron and colleagues also reported prevention and possibly reversal of pulmonary arterial remodeling with subsequent improvement in RV hemodynamics with spironolactone in monocrotaline rat models [106].

### 5.10. Genetics

Bone morphogenic protein receptor 2 (BMPR2) mutations are the most common genetic mutations noted in PAH. BMPR2 mutations have been described in around 80% of patients with hereditary PAH (hPAH) and about 20% patients with sporadic or idiopathic PAH [42,79,107]. The role of BMPR2 in PAH pathogenesis was initially advanced in early 2000 and 2001 with advances in whole-genome and whole-exome sequencing [42,108,109]. BMPR2 gene mutation is described to be “permissive of PAH” but requires an associated environmental and genomic second hit to develop in florid PAH endophenotype [9]. BMPR2 gene mutation has variable penetrance, averaging about 27% in patients with mutations. The penetrance, however, varies in different sexes with females (42%) having higher penetrance than men (14%) [9].

Loss of BMPR2 mediated signaling has a predominant role in PAH pathogenesis. BMPR2 plays a role in endothelial growth and functional loss of BMPR2 tips the scale towards a pro-proliferative and anti-apoptotic endophenotype [1,110] that has since been described as the pathognomonic molecular change in PAH. In addition, BMPR2 functional deficit in the endothelium also facilitated the mitochondrial metabolic dysregulation and inflammation [89]. Reduced BMPR2 levels have been noted in non-genetic animal models, thereby pushing for a role of BMPR2 target therapy in PAH treatment even in patients without mutations [9].

A multitude of newer therapeutic advances thus target BMPR2 signaling pathway. Altauren (PTC124) is an investigational drug that allows ribosomal read through of premature termination codons. Nearly 29% of hPAH patients have nonsense mutations in BMPR2 resulting in precocious stop codon and subsequent incomplete gene transcriptions and protein translations. Altauren has been proposed a potential treatment avenue for PAH by allowing for full length protein translation from mutated genes [111]. Research using HeLa cells transfected with mutant or wild type BMPR2 receptor constructs has also pointed towards a role of chaperone chemicals such thapsigargin, glycerol or sodium 4-phenylbutyrate, that permit increased trafficking of BMPR2 protein from endoplasmic reticulum and increased cell surface expression. These preclinical studies show a potential avenue for rescuing mutated BMPR2 and increased cell surface expression of the protein, which although mutated, is functional and shows ability to bind to BMP type 1 receptors [112]. A transcriptional luciferase reporter assay of 3756 FDA-approved drugs for induction of BMPR2 signaling by Spiekerkoetter et al. revealed best response with tacrolimus (FK506). Tacrolimus by binding to FKBP12, a suppressor for BMP signaling, allows for downstream SMAD1/5 and MAP kinase signaling. Authors also reported reversal dysfunctional BMPR2 signaling with low-dose tacrolimus in iPAH endothelial cells. Low dose tacrolimus also reverted severe PAH in monocrotaline rat models and hypoxia/VEGF (vascular endothelial growth factor) receptor blockade rat models [113]. Role of chloroquine and hydroxychloroquine in inhibiting autophagy and lysosomal degradation of BMPR2, with beneficial effects noted in experimental rat models have been reported as well [114].

Genomic analysis of PAH patients has also shown mutations in genes other than BMPR2. BMPR2 signaling pathway gene mutations described in PAH are ACVRL1, encoding ALK1, accessory receptor endoglin (ENG), and transcriptional mediators SMAD1, 4 and 9 mutations have been reported [110]. Exomic sequencing studies have also identified mutations in KCN3 and CAV1. KCN3 is responsible for encoding the pH sensitive potassium channel TASK1 [115]. CAV1 encodes caveolin 1 that forms lipid rafts or caveolae [116].

### 5.11. Epigenetic Mechanisms (miRNA, DNA Methylation and Histone Acetylation)

Epigenetic changes are modifications to DNA that alter and regulate gene expressions. These changes do not change the genomic sequence. The three major epigenetic changes identified to play a role in PAH pathogenesis are DNA methylation, histone modification and micro RNAs.

### 5.12. DNA Methylation

DNA methylation is an epigenetic change that results in gene silencing by preventing gene transcription. In PAH, increased expression of DNA Methyltransferases is noted in the lung endothelial cells and pulmonary artery smooth muscle cells. DNA methylation of superoxide dismutase (SOD) 2, results in SOD gene silencing. Up to 50% decline in SOD2 protein levels was observed in rat models with spontaneous PAH. SOD is an antioxidant defense against reactive oxidant species [117]. SOD in mitochondria converts the superoxide anion to hydrogen peroxide. Decreased SOD and consequently decreased hydrogen peroxide, results in upregulation of hypoxia inducing factor (HIF)-1α even in the absence of hypoxia. As described above, HIF-1α activation elicits Warburg effect that potentiates a *cancer like phenotype* with eventual smooth muscle cell proliferation and vasoconstriction [118]. Therapies targeting DNA methyltransferases, modulating demethylases, or using methyl binding proteins to alter gene transcription are future treatment options for PAH [9].

### 5.13. Histone Modification

Histone acetylation and deacetylation is an important post-translational modification regulating gene expression. Acetylation of histone allows for a loosely wound DNA that is more accessible to transcriptional machinery, thus permits gene expression. On the contrary, histone deacetylation leads to a more compactly wound DNA that is cannot be transcribed, and thus decreasing gene expression. The role of histone deacetylases (HDAC) in PAH has been noted in multiple animal model studies and from the promising results of small molecule inhibitors of histone deacetylases in these preclinical animal model studies [119].

Li et al. reported an increased HDAC catalytic activity and increased levels of HDAC 1, 2 and 3 in pulmonary adventitial fibroblasts in bovine model of hypoxia induced PH [120]. Role of valproic acid and vorinostat in rodent models of PAH was evaluated. Both drugs showed inhibition of the pro-proliferative phenotype [121]. Other inhibitors such as mocetinostat and entinostat have been shown to reduce pulmonary artery systolic pressure and improve arterial compliance in lungs in hypoxic rodent models [122]. Class II HDACs maintain myocyte enhancer factor (MEF) 2, a transcription factor with roles in cardiovascular development, in deacetylated state. Increased MEF 2 transcription has been reported in pulmonary artery endothelial cells in PAH and was thought to be mediated by increased concentration of HDAC 4 and 5 in the nucleus [123]. With that hypothesis, evaluation of inhibitors of Class IIa HDACs such as MC1568 has been reported to restore MEF 2 transcriptional activity with consequent resolution of proliferative phenotype [124]. However, subsequent in vitro analysis of MC1568 have failed to recreate the HDAC class II inhibition reported in prior studies [125].

### 5.14. Micro RNAs

Micro RNAs (miR) have been described to contribute to pathogenesis of metabolic dysregulation profile in PAH. miR non-coding RNA segments that silence gene expression by binding to the 3′ untranslated region on mRNA. Multiple miR have been reported to effect the PAH endophenotype, and they are emerging as newer therapeutic options (Table 3). MiR-126 and miR-21 were described to be upregulated in the plexiform lesions in PAH with miR-143/145 and miR-204 were noted to be higher in concentric PAH remodeling lesions [126].

In the setting of pulmonary artery hypertension and upregulation of interleukin 6 causes increase in signal transducer and activator of transcription (STAT) 3 and miR-17-92 cluster [127]. The latter has also been shown to be participant in BMPR2 related signaling affecting the proliferative endophenotype seen in BMPR2 dysregulation. It has been noted that miR-17-92 cluster is initially upregulated in hypoxic animal models initiating the PAH changes with cell proliferation and later downregulated in the later stages of the disease with decreased production of smooth muscle specific markers [81]. Inhibition of miR-17-92 cluster results in p21 induction that inhibits cell proliferation [128].

Elevated miR-138 and miR-25 have been implicated in mitochondrial metabolic dysfunction in PAH by downregulating MCU. Research studying the effects of nebulized miR-25 and miR-138 have reported upregulation and restoration of MCU levels and concurrent resolution of PAH proliferative endophenotype [86].

Elevated miR-143/145 levels have been noted in PAH and are thought to be in the setting of hypoxia. miR-143/145 cluster is responsible for smooth muscle differentiation-dedifferentiation regulation, with anti-miR-145 agents found to deter the PAH endophenotype in hypoxic animal models [129,130].

## 6. Conclusions

Pulmonary arterial hypertension is a complex disease process involving a myriad of genetic, molecular, and environmental factors acting in concert to produce the disease phenotype. While conventional therapies are available, they arguably target the vasoconstrictive aspects predominantly and have not shown a significant mortality benefit. The newly identified features of the disease such as the role of mitochondrial metabolic dysfunction, micro RNAs, and the rho-kinase and TGF-β pathways, all provide new therapeutic avenues for disease treatment. PAH is now increasingly seen as a multipronged disease involving multiple points of interaction between genetics, metabolomics, imbalance of vasoconstrictor and vasodilator responses, endothelial and smooth muscle dysfunction, thrombosis and platelet dysregulation, and mitochondrial and miRNA abnormalities. Further translational and clinical research is needed for the discovery of more efficacious clinical therapies in PAH.

## Figures and Tables

**Table 2:** Classification of pulmonary arterial hypertension (Modified and adapted from Simonneau et al., 2019 [30], Tang et al., 2016 [32]). PH—pulmonary hypertension, PAH—pulmonary arterial hypertension, PVOD—pulmonary veno-occlusive disease, PCH—pulmonary capillary hemangiomatous, LVEF—left ventricular ejection fraction, mPAP—mean pulmonary arterial pressure, PAWP—pulmonary arterial wedge pressure, PVR—pulmonary vascular resistance, WU—wood units, group 1—PAH, group 2—PH due to left heart disease, group 3—PH due to lung diseases and/or hypoxia, group 4—PH due to pulmonary arterial obstructions, group 5—PH with unclear and/or multifactorial mechanisms.

CLINICAL CLASSIFICATION OF PH		
PAH 1.1 Idiopathic PAH 1.2 Heritable PAH 1.3 Drug- and toxin- induced PAH 1.4 PAH associated with: 1.4.1 Connective tissue disease 1.4.2 HIV infection 1.4.3 Portal Hypertension 1.4.4 Congenital heart disease 1.4.5 Schistosomiasis 1.5 PAH long-term responders to calcium channel blockers 1.6 PAH with overt features of venous/capillaries (PVOD/PCH) involvement 1.7 Persistent PH of the newborn syndrome PH due to the left heart disease 2.1 PH due to heart failure with preserved LVEF 2.2 PH due to heart failure with reduced LVEF 2.3 Valvular heart disease 2.4 Congenital/acquired cardiovascular conditions leading to post-capillary PH PH due to lung diseases and/or hypoxia 3.1 Obstructive lung disease 3.2 Restrictive lung disease 3.3 Other lung disease with mixed/obstructive pattern 3.4 Hypoxia without lung disease 3.5 Developmental lung disorders PH due to pulmonary artery obstructions 4.1 Chronic thromboembolic PH 4.2 Other pulmonary artery obstructions PH with unclear and/or multifactorial mechanisms 5.1 Hematological disorders 5.2 Systemic and metabolic disorders 5.3 Others 5.4 Complex congenital heart disease		
WHO CLASSIFICATION OF PH		
I	Patients with pulmonary hypertension but without resulting limitations of physical activity. Ordinary physical activity does not cause undue fatigue or dyspnea, chest pain or heart syncope.	
II	Patients with pulmonary hypertension resulting in slight limitation of physical activity. They are comfortable at rest. Ordinary physical activity results in undue fatigue, or dyspnea, chest pain, or heart syncope.	
III	Patients with pulmonary hypertension resulting in marked limitation of physical activity. They are comfortable at rest. Less than ordinary physical activity causes undue fatigue or dyspnea, chest pain, or heart syncope.	
IV	Patients with pulmonary hypertension resulting in ability to carry on any physical activity without symptoms. These patients manifest signs of right heart failure. Dyspnea and/or fatigue may be present even at rest. Discomfort is increased by physical activity.	
HEMODYNAMIC CLASSIFICATION OF PH		
DEFINTIONS	CHARACTERISTICS	CLINICAL GROUPS
Pre-capillary PH	mPAP > 20 mmHg	
	PAWP ≤ 15 mmHg	1, 3, 4 and 5
	PVR ≥ 3 WU	
Isolated post-capillary PH	mPAP > 20 mmHg	
	PAWP > 15 mmHg	2 and 5
	PVR < 3 WU	
Combined pre- and post- capillary PH	mPAP > 20 mmHg	
	PAWP > 15 mmHg	2 and 5
	PVR ≥ 3 WU	

**Table 3:** Micro RNAs involved in pulmonary artery hypertension pathogenesis. miR: micro RNA, BMPR-II- type II: bone morphogenetic protein receptor; MCT: monocrotaline; NOS: nitric oxide synthase; PAEC: pulmonary artery endothelial cell; PASMC: pulmonary artery smooth muscle cell; PAH: pulmonary arterial hypertension; PH: pulmonary hypertension; NFAT: Nuclear factor activated T-cells; VEGF: Vascular endothelial growth factor; RV: Right ventricle; PPARΥ: Peroxisome-proliferator activated receptor gamma; MCU: Mitochondrial calcium uniporter; APLN: Apelin; FGF: Fibroblast growth factor; NA: Not applicable, ↑:Increased expression; ↓: Decreased expression.

MicroRNA	Expression in PAH	Human Model	Animal Model	Effect
miR-17-92	↑	NA	Mouse—Hypoxia Rat—monocrotaline, hypoxia	Increased PASMC proliferation, induced by IL-6; overexpression downregulates BMPR-II
miR-21	↑	Pulmonary arteries, plexiform lesions	Mouse—hypoxia, Sugen 5416/hypoxia, *VHL* null Interleukin-6 transgenic Rat—monocrotaline	Decreased NOS expression in hypoxic PAECs, increased PASMC proliferation; miR-21 deletion enhances PH in mice
miR-126	↓	Right ventricle	Rat—monocrotaline	Inhibition of VEGF pathway and decrease in RV vascular density
miR-145	↑	Lung tissue, plexiform lesions	Mouse—hypoxia, BMPR2 mutation	Decrease in miR-145 is protective against hypoxia-induced PAH
miR-150	↓	Plasma	NA	Associated with poor survival
miR-204	↓	Lung, pulmonary arteries	Rat—monocrotaline, Sugen5416/hypoxia Mouse—hypoxia	Increased NFAT, PASMC proliferation; miR-204 mimics prevent PH in monocrotaline model
miR-210	↑	Pulmonary artery	Mouse—Sugen5416/hypoxia	Inhibits PASMC apoptosis by suppressing E2F3 transcription factor expression
miR-214	↑	NA	Mouse—hypoxia, Sugen5416/hypoxia Rat—monocrotaline, Sugen5416/hypoxia	Increased right ventricular hypertrophy in hypoxia models
miR-130/301	↑	Pulmonary artery plasma	Mouse—hypoxia, Sugen5416/hypoxia, *VHL* null, Interleukin-6 transgenic, BMPR2X transgenic, *Schistosoma mansoni*—infected Rat—monocrotaline Juvenile lamb—pulmonary artery—aorta shunt	Increased PAEC proliferation and PASMC contraction via PPAR-Υ mediated pathways
miRNA-21 and miRNA-27a	↓	PAECs and PASMCs	NA	Suppress PAEC and PASMC proliferation
miR-26a	↓	PAH patient plasma	Rats—monocrotaline	Inhibition of miR-26a promotes apoptosis of rat cardiomyocytes and pathological right ventricular hypertrophy in PAH
miR-124	↓	Pulmonary artery smooth muscle cells	Mouse—chronic hypoxia	Suppression of NFAT pathway, antiproliferative
miR-138 and miR-25	↑	Pulmonary artery smooth muscle cells	Rats—monocrotaline	Downregulation of MCU, increased PASMC proliferation, apoptosis resistance; inhibition of miRs prevent PH in monocrotaline model
miR-140-5p	↓	NA	Rat—monocrotaline, Sugen-hypoxia	Inhibition of miR 140-5p promotes smooth muscle cell proliferation

## Data Availability

Not applicable.
